# Continuous Exposure to Non-Soluble β-Glucans Induces Trained Immunity in M-CSF-Differentiated Macrophages

**DOI:** 10.3389/fimmu.2021.672796

**Published:** 2021-06-02

**Authors:** Bart G. J. Moerings, Priscilla de Graaff, Matthew Furber, Renger F. Witkamp, Reno Debets, Jurriaan J. Mes, Jeroen van Bergenhenegouwen, Coen Govers

**Affiliations:** ^1^ Wageningen Food and Biobased Research, Wageningen University & Research, Wageningen, Netherlands; ^2^ Nutritional Biology Group, Division of Human Nutrition and Health, Wageningen University & Research, Wageningen, Netherlands; ^3^ Laboratory of Tumor Immunology, Department of Medical Oncology, Erasmus Medical Center (MC)-Cancer Institute, Rotterdam, Netherlands; ^4^ Danone Nutricia Research, Utrecht, Netherlands; ^5^ Cell Biology and Immunology Group, Department of Animal Sciences, Wageningen University & Research, Wageningen, Netherlands

**Keywords:** β-glucan, trained immunity, macrophage model, resilience, dectin-1

## Abstract

Beta-glucans enable functional reprogramming of innate immune cells, a process defined as “trained immunity”, which results in enhanced host responsiveness against primary (training) and/or secondary infections (resilience). Trained immunity holds great promise for promoting immune responses in groups that are at risk (*e.g.* elderly and patients). In this study, we modified an existing *in vitro* model for trained immunity by actively inducing monocyte-to-macrophage differentiation using M-CSF and applying continuous exposure. This model reflects mucosal exposure to β-glucans and was used to study the training effects of a variety of soluble or non-soluble β-glucans derived from different sources including oat, mushrooms and yeast. In addition, trained immunity effects were related to pattern recognition receptor usage, to which end, we analyzed β-glucan-mediated Dectin-1 activation. We demonstrated that β-glucans, with different sources and solubilities, induced training and/or resilience effects. Notably, trained immunity significantly correlated with Dectin-1 receptor activation, yet Dectin-1 receptor activation did not perform as a sole predictor for β-glucan-mediated trained immunity. The model, as validated in this study, adds on to the existing *in vitro* model by specifically investigating macrophage responses and can be applied to select non-digestible dietary polysaccharides and other components for their potential to induce trained immunity.

## Introduction

An important feature of the adaptive immune system is the ability to develop long-lasting memory responses. Over the past years, it has been clearly established that innate immune cells also retain a memory of previous challenges with the long-recognized hypo responsiveness following lipopolysaccharide challenges (LPS tolerance) and, the more recent established, hyper responsiveness following β-glucan challenges (trained innate immunity) (1, 2). These properties of innate immune cells have been termed ‘innate immune memory’ and are a consequence of long-term functional reprogramming of innate immune cells following an initial trigger (3). First indications for trained innate immunity in humans emerged from epidemiological studies on vaccine responses. In these studies, it was established that vaccination did not only lead to protection against a specific pathogen, but also to cross-protection against unrelated pathogens (4). The best described example is that of Bacillus Calmette-Guérin (BCG) vaccination against Mycobacterium tuberculosis, reducing neonatal mortality due to sepsis, respiratory infection, and fever (4). Next to offering cross-protection against pathogens, vaccination with BCG has also been shown to elicit anti-tumor immune effects. BCG vaccination reduced melanoma burden in adults but also associated with a reduced risk of developing melanomas in newborns (5–8). Preclinically, the pre-treatment of mice with a fungal β-glucan resulted in diminished tumor growth mediated through epigenetic effects on innate effector cells, providing evidence for the anti-cancer effects of immune training (9). These observations suggest that enhanced immune responses as a result of trained innate immunity might also be beneficial in immunotolerant states such as cancer.

Several studies have now shown that tolerance, a counter-regulatory mechanism to protect against collateral tissue damage in response to inflammation, can be reversed by induction of trained immunity to reinstate cytokine production upon re-challenge (10, 11). A study in healthy volunteers showed that β-glucan treatment resulted in enhanced responsiveness both by *in vivo* as well as *ex vivo* LPS-tolerized monocytes (11). Reversal of tolerance could be especially relevant for hospitalized elderly patients who often suffer from an attenuated immune response as a consequence of a previous insult, leading to increased susceptibility to secondary infections such as pneumonia (12). Recent data would suggest that supplementation of elderly with β-glucans could offer increased protection against the development of upper respiratory tract infections (13).

Trained innate immunity can be induced by a number of compounds containing danger-associated molecular patterns (DAMPs) or microbe-associated molecular patterns (MAMPs), such as oxidized low density lipoprotein (oxLDL), raw bovine milk and fungal cell wall derived β-glucans (13–15). In fact, yeast β-glucans are one of the best characterized stimuli to induce trained innate immunity (16, 17). Beta-glucans are large polysaccharides produced by a large variety of eukaryotic and prokaryotic organisms. All β-glucans share the same β-1,3-glucan backbone, however, the source and extraction methods determine the β-1,4 or β-1,6 branching patterns, insertions and impurities in the final commercial preparation (18, 19). While for some β-glucans such as oat (20), zymosan (21) and baker’s yeast (22) immune-modulatory activities have been described, for many others it is still unknown. The recognition of β-glucans by various immune cells is dependent on specific pattern recognition receptors (PRRs), which can initiate numerous downstream responses, including phagocytosis, respiratory burst, and secretion of cytokines and chemokines (23). Interestingly, immune cells derived from individuals deficient for the PRR Dectin-1 could not be trained with fungal β-glucans, which suggest that the C-type Lectin Dectin-1 is the primary candidate to confer innate immune training by β-glucans upon innate immune cells (24). Small intestinal routes of nutrient uptake can include capture by phagocytic immune cells through direct luminal sampling or the M-cell/Peyer’s patch route (25, 26). For β-glucans specifically, capture by gut-associated lymphoid tissue (GALT)-associated immune cells and/or epithelial cells has been demonstrated (27). Once internalized, β-glucan containing immune cells can travel to the different organs of the immune system were smaller β-glucan fragments are released over several days to further interact with immune cells via complement receptor 3 (CR3) and to modulate the functional capacity of immune cells (28, 29). These bioavailability studies were performed in mice and it remains to be determined whether it works similarly in humans. However, clinical efficacy data on β-glucan interventions and described effects on supporting innate immune functions would support a comparable mechanism (30).

The currently existing trained immunity model is not dedicated to investigate macrophage responses to continued oral exposure to β-glucans. In addition, limited β-glucans have been tested to provide insight into which physicochemical properties relate to induction of trained immunity. Therefore, we aimed to modify the current trained immunity model to reflect mucosal β-glucan exposure by introducing M-CSF-differentiated macrophages as well as continued exposure to β-glucans. To this end, we tested a broad panel of different commercially available dietary β-glucans for their potency to induce training and resilience. Metabolic and secretory markers of trained immunity were correlated to Dectin-1 activation with the aim to determine whether Dectin-1 activation could be a substitute for trained immunity in determining the potency of β-glucans.

## Materials and Methods

### Reagents

We used nine different β-glucans as previously described (31); yeast-a (Megazyme, Bray, Ireland), yeast-b (Immitec, Tonsberg, Norway), zymosan (InvivoGen, Toulouse, France), yWGP (InvivoGen) (all yeast-derived), curdlan [bacteria-derived, *Alcaligenes faecaeli*, (Megazyme)], lentinan [*Lentinula edodus* (31)], grifolan (*Grifola frondosa* [Hangzhou New Asia International Co., Ltd, Hangzhou, China)], schizophyllan [*Schizophyllum commune* (InvivoGen)] (all fungi-derived), and oatβG [oat-derived, (Megazyme)]. Their characteristics, such as solubility, protein content, molecular weight distribution, branching and linkages, monosaccharide composition, total saccharide content (*i.e.* purity) as well as LPS/LTA contamination levels have been reported previously (31).

### Isolation and Culture of Human Monocytes

Buffy coats from healthy donors were collected after written informed consent (Sanquin, Nijmegen, The Netherlands). Isolation of human peripheral blood mononuclear cells (PBMCs) was performed by dilution of the buffy coat fractions 1:1 with sterile phosphate-buffered saline (PBS) (Sigma Aldrich, Zwijndrecht, The Netherlands) containing 2% fetal bovine serum (FBS) (HyClone™ Fetal Bovine Serum, Fisher Scientific, Loughborough, UK) and loading onto Greiner Bio-One™ LeucoSEP™ Polypropylene Tubes that were pre-loaded with 15 ml Ficoll-Paque plus (GE Healthcare Life Sciences). Cells were centrifuged at 200xg for 5 min followed by centrifugation at 500xg for 10 min. The interface layer, containing PBMCs, was isolated and the cells were washed three times in PBS containing 2% FBS. After washing, cells were diluted in 8 ml MACS buffer (2 mM EDTA, 2% FBS in PBS), after which 1 ml of CD14 microbeads (Miltenyi Biotec, Leiden, The Netherlands) was added per buffy coat followed by an incubation step for 15 min at 4°C and mixing every 5 min. Cells were washed and resuspended in 0.5 ml MACS buffer, and monocytes were isolated using positive selection with the quadroMACS system and LS Columns according to the manufacturer’s protocol (Miltenyi Biotec). Sorted cells were frozen in FBS with 10% dimethyl sulfoxide (DMSO) (Sigma Aldrich) and stored in liquid nitrogen.

### Training and Resilience Model for Human Monocytes

**Figure 1:**
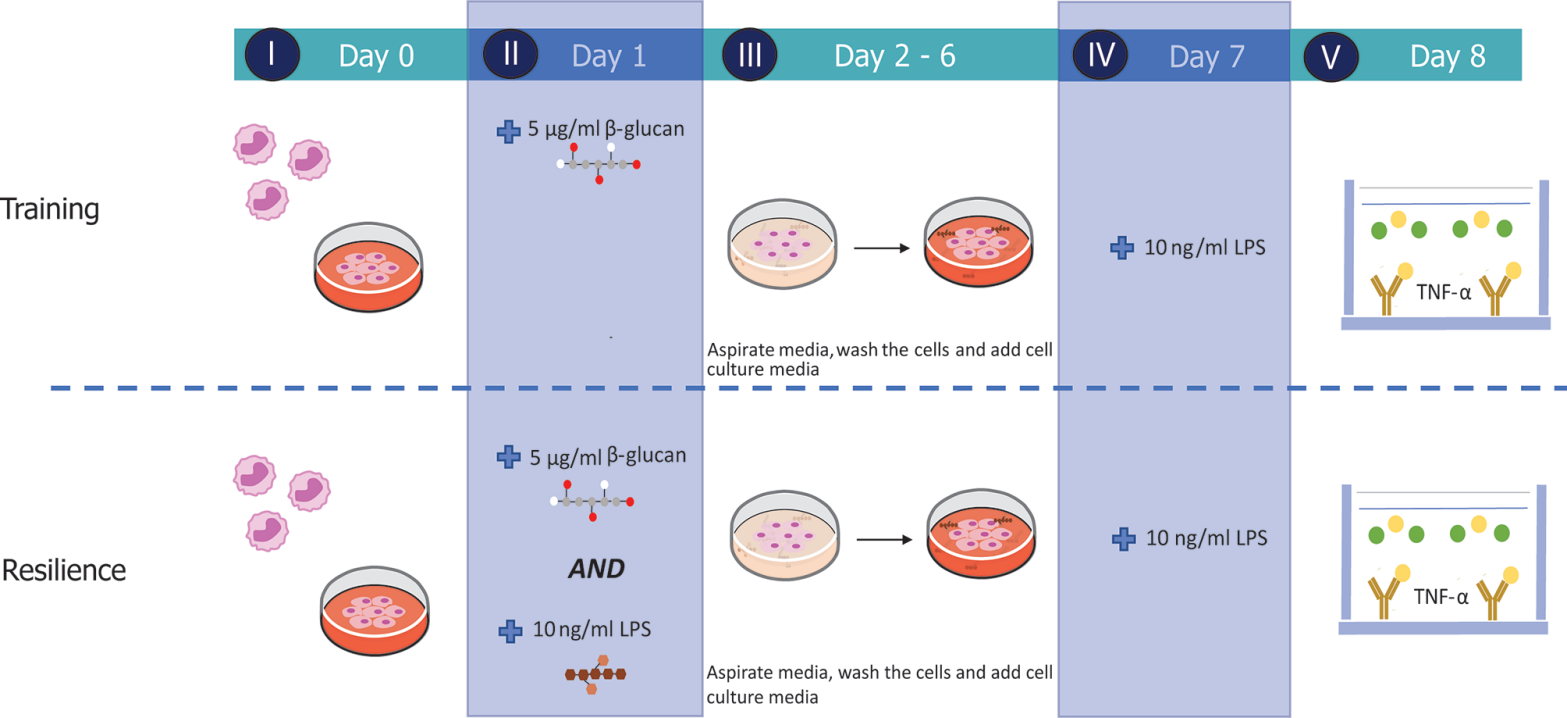
Experimental set-up of the established *in vitro* training and resilience protocols. Monocytes were retrieved from cryogenic vials and allowed to settle for 24 h (I) before cells were stimulated with medium, 5 µg/ml β-glucan (yeast-b, yWGP, grifolan), 10 ng/ml LPS or both β-glucan and LPS for 24 h (II). Stimuli were removed and cells were rested for 5 days (III) and subsequently challenged at day 7 with 10 ng/ml LPS (IV). Supernatant was collected on day 8 to quantify TNF-α levels measured by means of ELISA (V).

Monocytes (500,000 cells/well) were added to 24-well tissue culture (TC) plates (Corning Costar, New York, NY, USA) and incubated for 24 h at 37°C in RPMI 1640 – Glutamax – HEPES medium (Gibco, Bleiswijk, The Netherlands) supplemented with 10% FBS, 1% MEM with non-essential amino acids (Gibco), 1% Na-pyruvate (Gibco), 1% Pen/strep (Gibco) with or without 50 ng/ml macrophage colony-stimulating factor (M-CSF) (R&D systems, Minneapolis, MN, USA) in a total volume of 1 ml for 24 h at 37°C. Next, monocytes were stimulated by adding fresh medium, 5 µg/ml β-glucan, 10 ng/ml LPS (LPS derived from *Escherichia coli* O111:B4, Sigma Aldrich) or a combination of 5 µg/ml β-glucan and 10 ng/ml LPS with or without 50 ng/ml M-CSF (see **Figure 1**). After 24 h of stimulation, cells were washed once with pre-warmed medium, after which culture medium with or without 50 ng/ml M-CSF was added for another 5 days to start monocyte differentiation. At day 7, macrophages were stimulated for 24 h with 10 ng/ml LPS, after which the supernatant was collected and stored at -20°C for further analysis (see **Figure 1**). Alternatively, during the 5 day incubation period, monocytes were exposed to 5 µg/ml β-glucan and 50 ng/ml of M-CSF and the medium was collected and stored at -20°C for further analysis.

### Reporter Assay

The NFκB reporter cell lines HEK-Blue™Null1-v cells, HEK-Blue™-hDectin-1a and HEK-Blue™-hDectin-1b (InvivoGen) were cultured and maintained in high glucose DMEM GlutaMAX™ (Gibco) supplemented with 10% heat-inactivated FBS (Gibco). These reporter cell lines overexpress the Secreted Embryonic Alkaline Phosphatase (SEAP) reporter gene driven by an NFκB-inducible promoter. All reporter assays were performed according to the manufacturer’s protocol. Briefly, cell passage was performed by trypsinization with 0.05% trypsin-EDTA (Life Technologies) and a split ratio of 1:10 was used. All cell lines (passage 4-32) were seeded at 1x10^6^ cells/ml in 100 µl/well in a poly-D-Lysine coated 96-well microplate (Greiner bio-one, Alphen a/d Rijn, The Netherlands) overnight at 37°C and 5% CO_2_. The following day, reporter cell lines were stimulated for 24 h with different concentrations of β-glucans (5, 10, 100 and 1000 µg/ml) in a total volume of 200 µl/well, after which, cell-free volumes of 20 µl/well were transferred to a 96 well-plate (Corning Costar) containing 180 µl/well QUANTI-Blue™ Solution (InvivoGen). Following a last incubation of 2 h at 37°C and 5% CO_2_, SEAP secretion was measured spectrophotometrically at 635 nm (TECAN, Giessen, The Netherlands).

### Cytokine Production

The production of interleukin (IL)-6 and tumor necrosis factor alpha (TNF-α) in cell-conditioned supernatants was determined by means of ELISA (BioLegend, San Diego, CA, USA) according to manufacturer’s protocol.

### Nitric Oxide Production

To measure nitric oxide (NO) production, monocytes were trained for 5 days with β-glucans and NO was determined in supernatants on day 6 before stimulation with LPS using Griess reagent (Sigma-Aldrich).

### Lactate Release

To assess release of lactate, monocytes were again trained for 5 days with β-glucans. After stimulation with LPS on day 7, supernatants were collected. The lactate concentration in supernatant was measured using a lactate colorimetric assay kit (Bio-connect, Huissen, The Netherlands).

### Statistical Analysis

All experiments were conducted with a minimum of five human donors. All data were analyzed using GraphPad Prism software version 5.0 **(**Graphad, La Jolla, CA, USA). Results were analyzed using a paired Student’s t-test, one-tailed Spearman’s correlation test or 2-way ANOVA. A *P* value <0.05 was considered as statistically significant. Data are shown as means **±** standard deviation (SD).

## Results

### Yeast-Derived β-Glucan Induced Training and Resilience in M-CSF-Differentiated Macrophages

**Figure 2:**
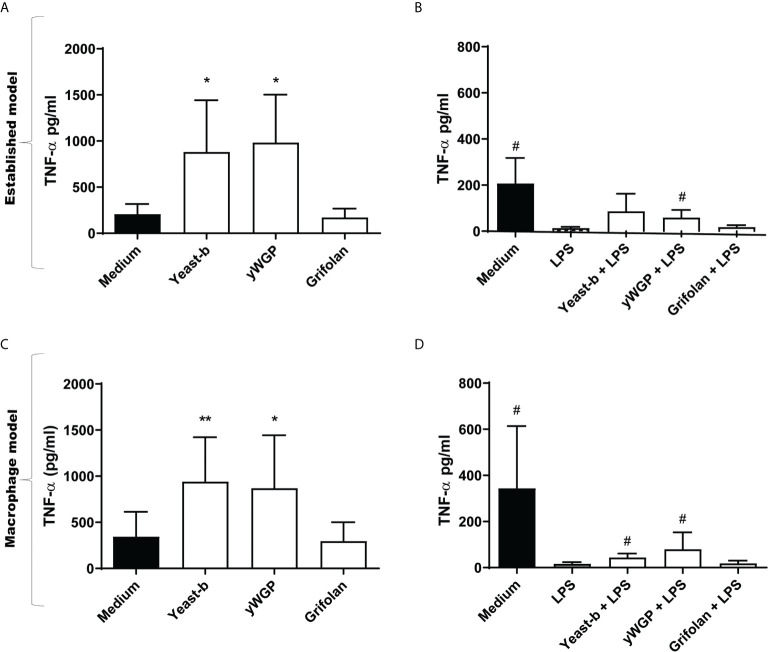
Beta-glucans induced training and increase resilience in both trained immunity protocols. The protocols of the *in vitro* training and resilience model for **(A–D)** are schematically depicted in **Figure 1** and described in the legend to **Figure 1**, with the adjustment that 50 ng/ml M-CSF was added to step I-III for **(C)** and **(D)** Results from training **(A)** and induced resilience **(B)** when applying the established protocol and training **(C)** and induced resilience **(D)** when applying the controlled M-CSF-mediated macrophage differentiation protocol are shown in a bar graph as average pg/ml TNF-α ± SD of *n* = 5 different donors. Data was analyzed with paired Student’s *t* test and statistical significances were indicated: *P < 0.05 compared to the medium control; **P < 0.01 compared to the medium control; ^#^P < 0.05 compared to the LPS control.

We investigated whether a yeast-derived WGP (yWGP), extensively studied for its immunomodulatory activities, a second yeast-derived β-glucan extract (yeast-b) and a third fungi-derived β-glucan (grifolan) induce training or resilience when applying the established model as depicted in **Figure 1**. To control macrophage differentiation, the established model (16) was modified by adding M-CSF at all steps and using FBS instead of human serum. Yeast-b and yWGP, but not grifolan, enhanced release of TNF-α following an LPS stimulus at day 7 in both the established and macrophage training models (**Figures 2A, C**). Beta-glucan yWGP also increased TNF-α release when compared to LPS-induced tolerance in the resilience protocol using either models while yeast provided resilience only in the adapted model and grifolan was unable to increase TNF-α release compared to LPS-induced tolerance in either model (**Figures 2B, D**).

### Yeast-b Induces Training and Resilience in M-CSF-Induced Macrophages Following Continuous Exposure

**Figure 3:**
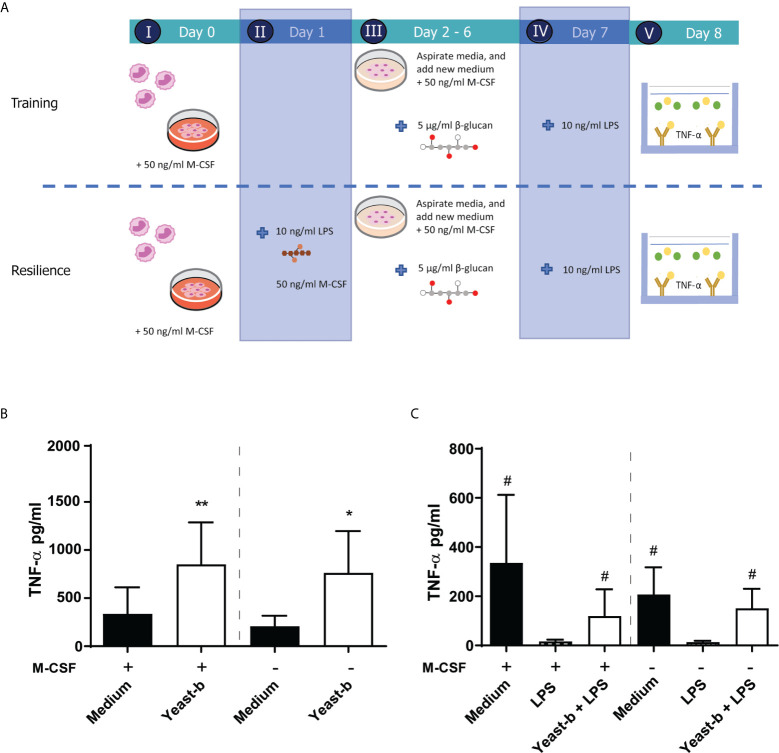
Introducing new elements to the established training and resilience protocol to generate a macrophage model system. The protocol of *in vitro* training and induced resilience including the addition of M-CSF during step I-III and a prolonged incubation (*i.e.*, day 2 to 6 instead of day 1) with β-glucan in the macrophage model is depicted in **(A)** Results from training **(B)** and induced resilience **(C)** in presence or absence of M-CSF and prolonged presence of β-glucan are shown in a bar graph as average pg/ml TNF-α ± SD of *n* = 5 different donors. Data was analyzed with paired Student’s *t* test and statistical significances were indicated: *P < 0.05 compared to the medium control; **P < 0.01 compared to the medium control; ^#^P < 0.05 compared to the LPS control.

To mimic daily intake of β-glucans, we investigated the effect of continuous exposure by supplementing β-glucans to day 2 until day 6 cultures without medium refreshment, as schematically depicted in **Figure 3A**. Again, at day 1 the LPS challenge was provided in the absence of β-glucans, as β-glucans can bind LPS, and therefore could prolong LPS presence in the medium (32). Similarly, as observed with single day exposure, continuous exposure in both the established and M-CSF modified training models enhanced release of TNF-α following an LPS-stimulation on day 7 (**Figure 3B**). Continuous exposure of LPS-activated cells to yeast-b enhanced the release of TNF-α following a secondary LPS stimulation on day 7 compared to cells receiving LPS as primary stimulus and did not receive yeast-b (**Figure 3C**). Taken together, our macrophage model of controlled and M-CSF-mediated macrophage differentiation and continuous exposure to β-glucans can demonstrate the induction of trained immunity by yeast-b.

### Yeast and Bacteria-Derived β-Glucans Enhanced Secretion of Pro-Inflammatory Cytokines IL-6 and TNF-α in Training and Resilience Protocols With M-CSF-Induced Macrophages

With our macrophage model of M-CSF addition and continuous exposure to β-glucans, we next evaluated a range of different soluble and insoluble β-glucans derived from different sources for their effect on training and resilience. Next to yeast-b, yWGP and grifolan, we tested yeast-a, zymosan, curdlan, lentinan, oatβG and schizophyllan. All were previously characterized for their physicochemical composition and LPS/LTA contamination levels (31).

**Figure 4:**
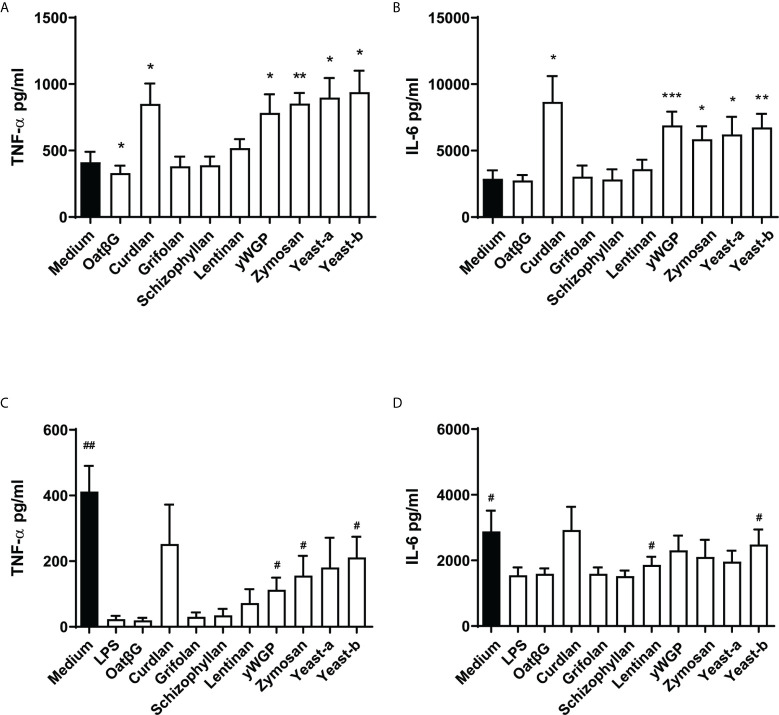
Yeast-b, zymosan, curdlan, yWGP and yeast-a induced training and/or resilience in the macrophages model with continuous exposure. Monocytes were treated as depicted in **Figure 3A**. Monocytes were exposed to medium from days 1 to 6, or trained with 5 μg/ml β-glucans from days 2 to 6, before testing the training effect by challenging them with 10 ng/ml LPS at day 7 for 24 h **(A, B)**. Alternatively, to test induced resilience, monocytes were challenged with LPS at day 1, from days 2 to day 6 cells were cultured in medium or with 5 μg/ml β-glucans and challenged with 10 ng/ml LPS at day 7 for 24 h **(C, D)**. Supernatants of conditioned media were collected and TNF-α **(A, C)** and IL6 **(B, D)** measured by ELISA. Results are shown in a bar graph as average pg/ml cytokine ± SD of *n* = 6 different donors. Data was analyzed with paired Student’s *t* test and statistical significances were indicated: *P < 0.05 compared to the no β-glucan (medium) control; **P < 0.01 compared to the medium control; ^#^P < 0.05 compared to the LPS control. ***P < 0.001 compared to the medium control.

Upon employing the training protocol, supplementation with yeast-b, zymosan, curdlan, yWGP or yeast-a, all β-glucans increased TNF-α and IL-6 release compared to medium control, while lentinan, grifolan, oatβG and schizophyllan showed no additional TNF-α or IL-6 release (**Figures 4A, B**). OatβG even showed a reduction in TNF-α release compared to medium control. In cultures receiving a primary trigger with LPS (*i.e.*, resilience protocol), supplementation with yeast-b, zymosan and yWGP increased TNF-α release following a secondary LPS triggering compared to the LPS control (**Figure 4C**). The observed effect was less strong for IL-6, with only yeast-b and lentinan showing a significantly increased release of IL-6 compared to the LPS control (**Figure 4D**). Supplementation with curdlan also increased TNF-α and IL-6 releases, however, these releases did not reach statistical significance.

### Beta-Glucans From Variable Sources Activated Dectin-1a and -1b Receptor Isoforms in a Concentration-Dependent Manner

**Figure 5:**
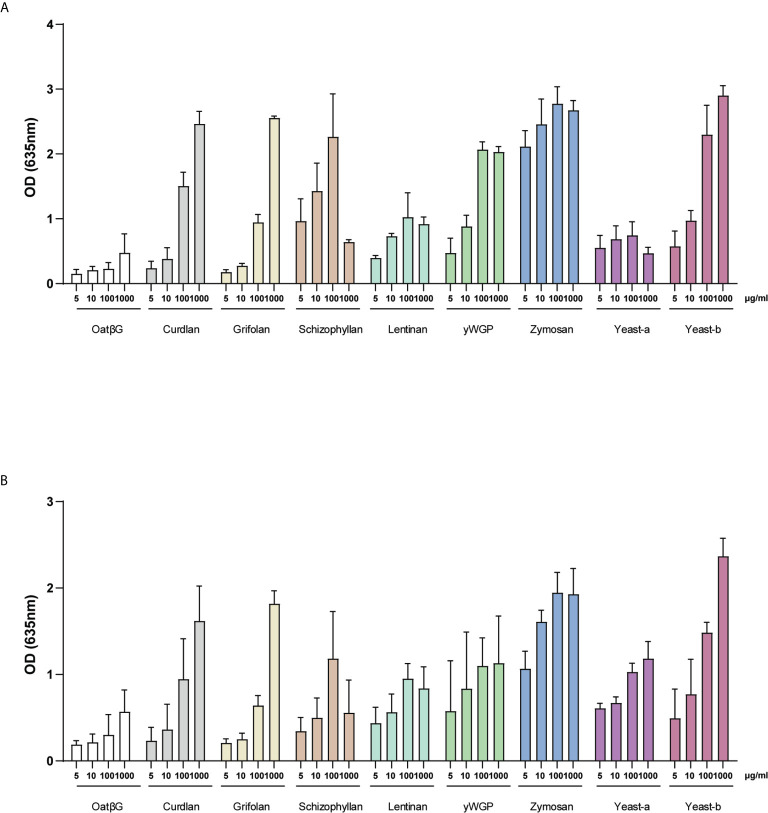
Activation of Dectin-1a and b isoforms by yeast-b, zymosan, curdlan, yWGP, lentinan, schizophyllan and yeast-a. HEK- Blue™ - Dectin-1a **(A)** and HEK- Blue™ - Dectin-1b **(B)** were stimulated with 5, 10, 100 and 1000 µg/ml of β-glucans. After 24 h of stimulation, the secretion of SEAP was quantified in cell-free supernatants. Results of SEAP activity analysis are shown in a bar graph as average values ± SD of absorbance at 635 nm from n = 3 independent experiments.

Dectin-1 is a primary receptor for β-glucans to exert their immunomodulatory activity. To identify whether the β-glucans that were tested in this study can also signal via this receptor, the activation of both Dectin-1 isoforms (*i.e.*, 1a and 1b) was measured. HEK-Blue Dectin-1 reporter cells and their controls were stimulated with 5, 10, 100 and 1000 µg/ml of β-glucans for 24 h and their activation measured as described in the materials and methods. Both Dectin-1a (**Figure 5A**) and Dectin-1b (**Figure 5B**) transfected cells, but not the non-transfected control cell line Null-1V (**Supplementary Figure 1**), showed activation following β-glucan supplementation. When using a high dose of 100 and 1000 µg/ml, all β-glucans, except oatβG and yeast-a, showed concentration-dependent activation of both Dectin-1a and Dectin-1b. Of note, schizophyllan appeared to lower the activation of both Dectin-isoforms when applying the highest dose. At a dose of 5 and 10 µg/ml, the differences between β-glucan preparations became more noticeable revealing yeast-b, zymosan, yWGP, schizophyllan and yeast-b as the most potent inducers of Dectin-1a and Dectin-1b activation.

### Dectin-1b Activation by β-Glucans Correlated With Lactate and TNF-α Secretion Following Training of Macrophages

**Figure 6:**
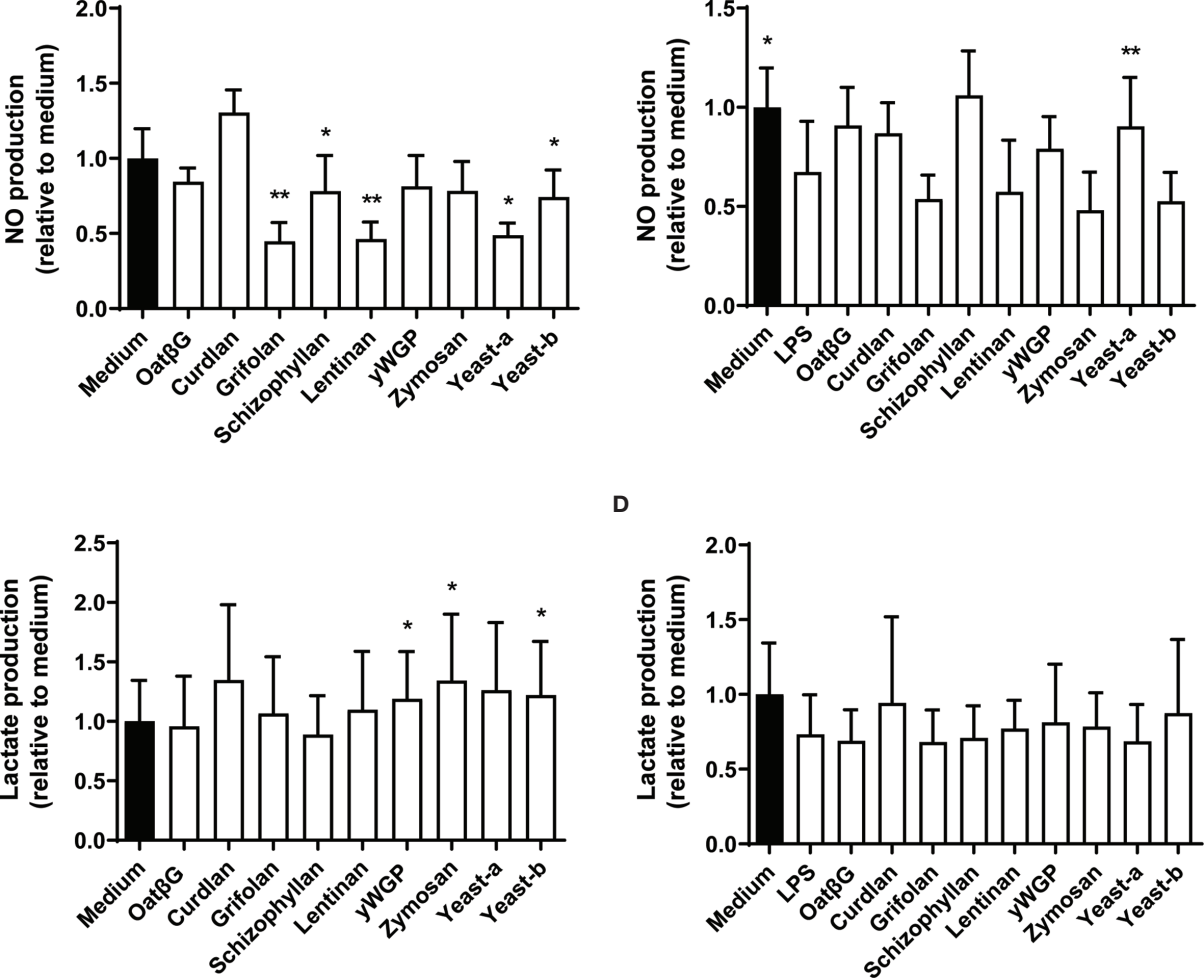
The induction of training and resilience in macrophages by various β-glucans affects NO and lactate secretion. Human monocytes were trained **(A, C)** and resilience was induced **(B, D)** as described in the legend to **Figure 3**. Nitric oxide **(A, B)** was determined before challenging the macrophages with LPS at day 7 and lactate **(C, D)** release was determined after day 7 of LPS challenge. NO and lactate levels were determined in the supernatant and shown as percentages relative to non-treated macrophages (medium is set at 100%). Results are shown in bar graphs as average ± SD of *n* = 3-5 different donors. Data was analyzed with paired Student’s *t* test and statistical significances were indicated: *P < 0.05 compared to the no β-glucan (medium) control; **P < 0.01 compared to the medium control.

To study the functional consequence of β-glucan-induced immune training of macrophages in more detail, we investigated the release of nitric oxide (NO) and lactate. In both experimental protocols (training / resilience) NO release was assessed before the LPS challenge at day 7 while lactate release was measured 24 h after challenge with LPS at day 7. Nitric oxide secretion was non-significantly increased in macrophages supplemented with curdlan, and significantly reduced in macrophages supplemented with grifolan, schizophyllan, lentinan, yeast-a or yeast-b, when compared to medium only (**Figure 6A**). In cultures receiving a primary trigger with LPS on day 1, yeast-a significantly increased NO secretion when compared to medium following a secondary LPS challenge on day 7 (**Figure 6B**). Macrophages supplemented with yWGP, zymosan or yeast-b resulted in an increased concentration of lactate in their medium when compared to medium treated macrophages (**Figure 6C**). Macrophages receiving an LPS-challenge on day 1 followed by supplementation with β-glucans demonstrated no significant change in lactate secretion when compared to medium following a secondary LPS challenge on day 7 (**Figure 6D**).

**Figure 7:**
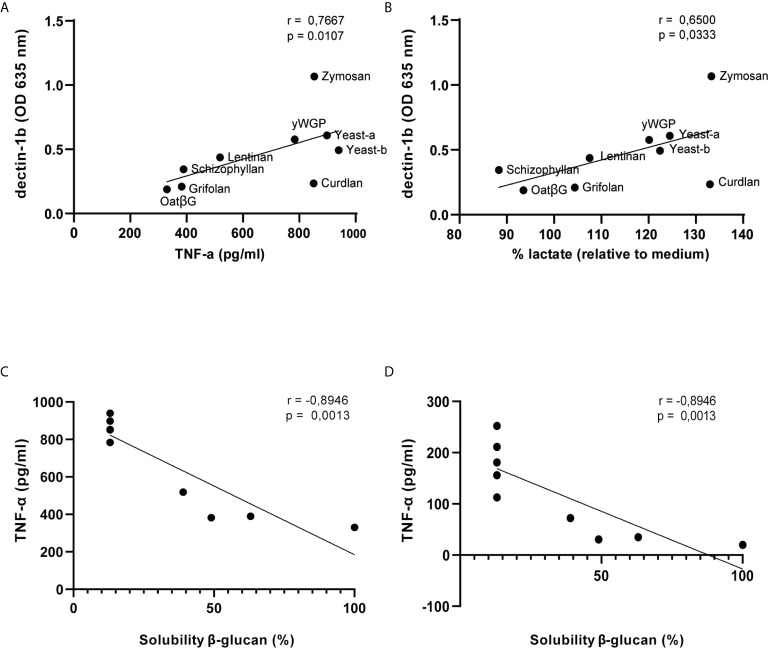
Dectin-1b activation and solubility of β-glucans correlate with macrophage functions following training or induced resilience. Scatterplots displaying SEAP release upon exposure of 5 μg/ml β-glucans to HEK- Blue™ - Dectin-1b cells and TNF-α **(A)** or lactate **(B)** release following training of macrophages. Correlation coefficients of Dectin-1b activation with macrophage secretion of TNF-α and lactate upon exposure of 5, 10, 100 and 1000 μg/ml β-glucans are displayed in **Supplementary Table 1**. Correlations between β-glucan solubility and TNF-α release following training **(C)** or resilience **(D)** testing in macrophages. Solubility of β-glucans was measured previously by de Graaff and colleagues (31): oatβG (100%), curdlan (<13%), grifolan (49%), schizophyllan (63%), lentinan (39%), yWGP (<13%), zymosan (<13%), yeast-a (<13%) and yeast-b (<13%).

Finally, we tested whether Dectin-1 activation correlated with above mentioned macrophage functions (**Supplementary Table 1**). In the training protocol, correlations found between secreted molecules and Dectin-1b were more significant rather than with Dectin-1a activation. Notably, TNF-α release, but not IL-6, was found to correlate with Dectin-1b across all β-glucan concentrations (**Figure 7A** and **Supplementary Table 1**). Nitric oxide release and Dectin-1 activation showed no correlation. Lactate release correlated with Dectin-1b activation for β-glucan concentrations of 5, 10 and 1000 µg/ml (**Figure 7B** and **Supplementary Table 1**). In the resilience protocol correlations between macrophage functions and Dectin-1 activation was far less evident. Only a few correlations were found at the highest β-glucan concentrations for both Dectin-1 isoforms. At a dose of 1000 μg/ml Dectin-1a and Dectin-1b activation correlated with NO and/or IL-6, and at a dose of 100 μg/ml Dectin-1a activation correlated with lactate release. Of interest, solubility, which is considered a defining feature in non-digestible polysaccharide functionality (33), significantly correlated with TNF-α in both protocols (**Figures 7C, D**). In addition, solubility of β-glucans also significantly correlated with IL-6 in both protocols (data not shown).

## Discussion

There is increasing evidence that β-glucans from different sources can modulate local and systemic immune responses. Data of our study clearly underline that β-glucans possess the ability to induce trained immunity and resilience of the innate immune system. However, several knowledge gaps remain regarding their mechanisms of action and structure-activity relationships. For instance, in human clinical studies it is not clear whether β-glucans can also be sufficiently absorbed from the intestinal tract, as Leentjes and colleagues found hardly detectable serum concentrations of β-glucan in blood (34). In addition, Lehne and colleagues found low concentrations of serum β-1,3 glucan without detection of systemic absorption following oral administration (35).

In contrast, preclinical studies have shown that orally ingested β-glucans can be absorbed in the gut (27, 28), in particular by macrophages, rather than monocytes, that are present in the proximal small intestine and are subsequently transported to distant lymph nodes, bone marrow and spleen (28). The existing immune training model relies on human serum with unknown and variable levels of monocyte-differentiation factors to induce macrophages from monocytes. To remove this variability, we opted to use M-CSF at a concentration known to yield macrophages (36). Our findings indicate that both models show similar induction of both innate immune training and resilience as measured by the increased release of TNF-α and IL-6 following supplementation with β-glucan. A limitation of the current models is that both serum and M-CSF-differentiated monocyte-derived macrophages may not fully mimic the function of intestinal tissue resident macrophages (TRMs), that will probably ligate, engulf and fragment orally ingested β-glucans after intake. Tissue-derived macrophages originating from different sources were demonstrated to share over 90% similarity in their genetic repertoire with only limited phenotypic differences, suggesting that the tissue micro-environment plays an important role in directing macrophage functionality (37–40). To improve the predictive value of the current immune cell models, future studies should focus on enhancing environmental mimicry through, for instance, the combination with intestinal organoids.

We next considered the exposure strategy for the therapeutic or preventive supplementation of β-glucans to support host immunity by replacing the single intake approach with daily intake. So, we made another change to the established model by providing continuous exposure instead of a single exposure. Measuring the effect of β-glucan supplementation in both models revealed that both the established model as well as the macrophage model led to enhanced training as well as resilience. Collectively, our macrophage model reflects mucosal exposure to β-glucans via the oral route and can be used to screen β-glucans for their training and resilience effects in a similar fashion as the established model.

In our validated macrophage model, multiple β-glucans from a variety of sources, with reported purity and solubility, were screened for induction of training or resilience. Results are in line with literature in that none of the soluble β-glucans, not even the high molecular weight fraction of such β-glucans, induced TNF-α or IL-6 production in either human monocytes or monocyte-derived macrophages (33). In contrast with our findings, Pan and colleagues showed that oat-derived β-glucan induces trained immunity through metabolic reprogramming (20). The oat-derived β-glucan used in our studies was highly soluble and induced neither immune training, resilience nor Dectin-1 activation. A likely explanation for this discrepancy could be a difference in the preparation of the tested oat-derived β-glucans, with the preparation used in the study by Pan and colleagues potentially containing an insoluble component. Another explanation could be the difference in cell origins as Pan and colleagues employed a monocytic cell line and mouse bone-marrow derived monocytes. Since PPR interaction by β-glucans are critical for innate immune training, differential PRR expression between human primary cells versus mouse immune cells, or human cell lines for that matter, may further underlie the different findings between the two studies. In fact, mouse and human Dectin-1 do not similarly mediate β-glucan recognition nor activation (41) and while primary monocytes and macrophages constitutively express Dectin-1, the human THP-1 cell line needs to be activated to do so (42, 43). In addition to analyze secretion of IL-6 and TNF-α, we also assessed the induction of NO and lactate secretion. Training of monocytes was shown to induce a shift in the metabolism from oxidative phosphorylation toward aerobic glycolysis (44) with lactate being a marker for glycolysis (10, 16). Using our macrophage model, we validated the metabolic shift towards increased glycolysis by showing increased lactate release, associated with increased cytokine production. In contrast, the release of NO was not altered as a result of training or resilience induction by β-glucans. This was also in line with previous findings (16, 45).

To the best of our knowledge, we demonstrated for the first time that human Dectin-1b activation by β-glucans was significantly and positively correlated with TNF-α secretion and lactate production following training of macrophages. In accordance with these results, previous studies demonstrated that maturation of monocytes was accompanied by enhanced expression of the Dectin-1b isoform, whereas isoform 1a expression tended to decline during maturation (46). Moreover, differentiation of monocytes with M-CSF also increased expression of Dectin-1b isoform that mainly conveys signals through the Syk/cPLA2 route (47). In the present study, particulate β-glucans demonstrated strong and significant activation of Dectin-1a as well as Dectin-1b receptors, also observed for some soluble β-glucan at high concentrations. It is noteworthy that insolubility overall strongly correlated to TNF-α release in both training and resilience assays. Goodridge and colleagues demonstrated that soluble β-glucans could reduce particulate β-glucan activity (33). They reported that, despite efficient binding to Dectin-1, soluble β-glucans are incapable of activating this receptor, and their presence might explain the lower Dectin-1 activation as we observed for schizophyllan specifically at a high concentration. Furthermore, these authors showed that immobilization of soluble β-glucans allows for Dectin-1 activation potentially due to the formation of a phagocytic synapse. However, the paper by Goodridge and colleagues does not indicate whether they investigated Dectin-1a or Dectin-1b. Although the differences between Dectin-1a and Dectin-1b function remains an understudied aspect of Dectin-1 function, literature would suggest that ligand binding might be different between Dectin-1a and Dectin-1b isoforms (48). Moreover, both isoforms are differently susceptible to neutrophil elastase cleavage which could impact the immune response towards pathogens (49). This could indicate that Dectin-1a or Dectin-1b usage might offer a means to regulate cellular responses towards β-glucan. Our own findings suggest that soluble β-glucans are capable of inducing Dectin-1 receptor clustering on HEK cells when applied at high enough concentrations. This, together with the observed correlations between Dectin-1b activation and macrophage functions, argues that Dectin-1b-expressing HEK cells may represent a pre-screening tool to assess the ability of compounds to train macrophages. In addition to Dectin-1, the CR3 receptor has also been demonstrated to be necessary to induce training by β-glucans (24). In fact, blocking of Dectin-1 or CR3 inhibited the priming of monocytes by β-glucans (23). Therefore, extending the mentioned pre-screening with a cell line to also identify CR3 activation is considered a valuable addition.

Finally, we would like to state that our model is limited to assess direct effects on macrophages, while the translation to the human *in vivo* situation will be more complex. Data have shown that the Dectin-1 receptor is essential for the distribution of β-glucans by macrophages through the body (50). After ingestion, β-glucans are slowly degraded and smaller β-glucan fragments are systemically released to be recognized by and activate CR3 present on various innate immune cells (24, 50). Together these lead to increased responsiveness of the innate immune response upon secondary challenges. It is still a matter of debate whether primary activation of Dectin-1 is necessary and whether β-glucan binding and uptake is sufficient for the *in vivo* effect of β-glucans. Findings presented in this study and reports on *in vitro* data clearly indicate a role for β-glucan solubility in the induction of immune innate training. Although blocking of CR3 was shown to only partially prevent induction of immune training (24), future studies should aim to investigate the activation of CR3 or other PRRs by β-glucan fragments to provide additional proof whether or not Dectin-1-mediated uptake of β-glucans is sufficient to induce immune training. Importantly, β-glucans were shown to reduce tumor onset, growth and progression in murine models (51). However, β-glucans from different molecular sizes, soluble/insoluble ratios and branching patterns may have significantly variable bioavailability and immune potency, and consequently variable anti-tumor effects. Therefore, the applied model for β-glucan selection must fit and represent the intended *in vivo* activity to accurately investigate clinical effects of β-glucans.

In conclusion, firstly this study presents a new macrophage-based *in vitro* model that reflects mucosal immunomodulation by β-glucans. We have shown that indigestible β-glucans induce training and/or resilience in macrophages leading to an increase in cytokine production and glycolysis. Furthermore, soluble and particulate β-glucans demonstrated different effects on Dectin-1 activation, which might be an additional explanation for the difference in trained innate immunity effects of soluble and particulate β-glucans as previously found by others.

Secondly, our data suggest that both insolubility and Dectin-1b activation predict whether β-glucans contain the capacity to induce training or resilience. Therefore, physicochemical analysis as well as Dectin-1b ligation could be considered a proxy for induction of training or resilience in macrophages, which in turn yield a simplified model to identify dietary ingredients with macrophage training properties. A simplified model to screen dietary fibers, beyond β-glucans, for their potential to induce immune training would benefit manufacturers who would aim to include immune-potentiating ingredients in their products.

## Data Availability Statement

The raw data supporting the conclusions of this article will be made available by the authors, without undue reservation.

## Author Contributions

BM and PG acquired the data. BM and PG analyzed and interpreted the data. BM and PG drafted the manuscript. CG, JB, RW, RD, JM, and MF critically revised the manuscript. All authors contributed to the article and approved the submitted version.

## Funding

This research is funded by the Partnership between NWO domain Applied and Engineered Sciences and Danone Nutricia Research and with additional financial support from Topsector Agri and Food. Danone Nutricia Research was not involved in the study design, collection, analysis, interpretation of data, the writing of this article or the decision to submit it for publication. This work was further supported by the Dutch Cancer Society [WUR 2015–7734] ‘Food-derived beta-glucans and fungal proteins to support anti-melanoma immune therapy’ and by the Dutch Ministry of Economic Affairs (KB-23-001-015).

## Conflict of Interest

MF and JB are employed by Danone Nutricia Research.

The remaining authors declare that the research was conducted in the absence of any commercial or financial relationships that could be construed as a potential conflict of interest.
