# Supplementary material for: Continuous Exposure to Non-Soluble β-Glucans Induces Trained Immunity in M-CSF-Differentiated Macrophages
Source: Front Immunol. 2021 Jun 2;12:672796. doi: 10.3389/fimmu.2021.672796 (PMC8208035; doi:10.3389/fimmu.2021.672796)
Supplement: Supplementary file 1 [file DataSheet_1.pdf]

Supplementary data

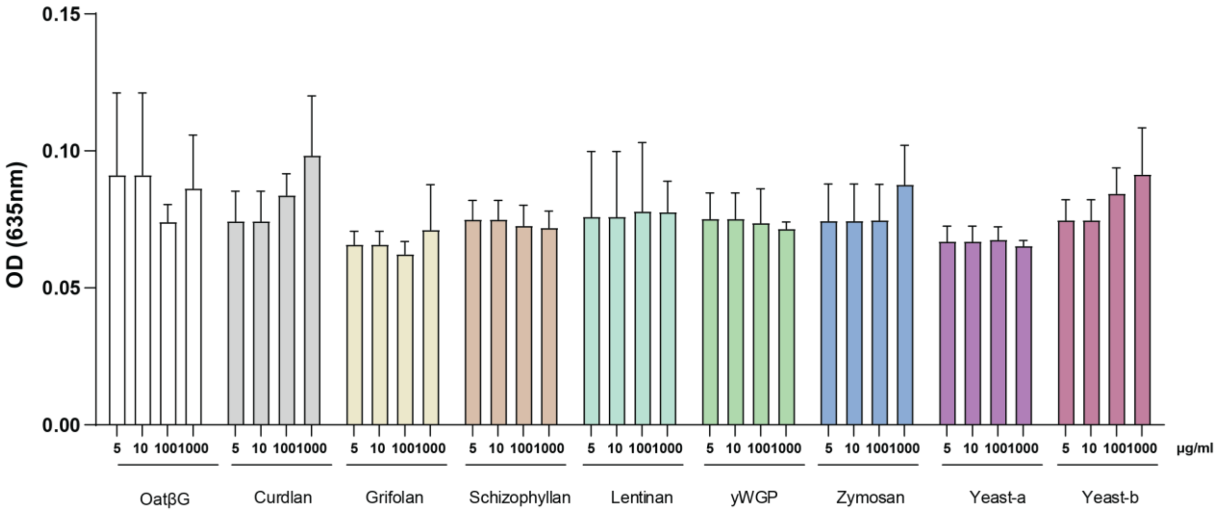

Supplementary Figure 1. Beta-glucans do not activate the parental cell-line Null1-v that lacks both dectin-1 isoforms. The parental cell line Null1-v cells were stimulated with 5, 10, 100 or 1000 µg/mL of β-glucans. After 24h stimulation, the secretion of SEAP was quantified in cell-free supernatants. Results of SEAP activity analysis are shown in a bar graph as average values ± SD of absorbance at 635 nm from n = 3 independent experiments.

Supplementary Table 1. Correlation of dectin-1a or -1b activation with macrophage secretion of TNF-α, IL-6, lactate or NO.

Model	Conc. β-glucan (µg/ml)	p-value	r	Read-out parameter/receptor
Training	5	0,1832	0,3218	IL-6/Dectin-1a
	10	0,3218	0,1833	
	100	0,1793	0,3500	
	1000	0,1563	0,3833	
	5	0,1063	0,4667	IL-6/Dectin-1b
	10	0,0809	0,5167	
	100	0,2183	0,3000	
	1000	0,0809	0,5167	
	5	**0,0429**	0,6167	TNF-α/Dectin-1a
	10	0,0888	0,5000	
	100	0,0888	0,5000	
	1000	0,1681	0,3667	
	5	**0,0107**	0,7667	TNF-α/Dectin-1b
	10	**0,0156**	0,7333	
	100	**0,0294**	0,6667	
	1000	**0,0333**	0,6500	
	5	0,1793	0,3500	Lactate/Dectin-1a
	10	0,2467	0,2667	
	100	0,1681	0.3667	
	1000	0,1348	0,4167	
	5	**0,0333**	0,6500	Lacate/Dectin-1b
	10	**0,0484**	0,6000	
	100	0,1456	0,4000	
	1000	**0,0216**	0,7167	
	5	0.4216	-0.0833	NO/Dectin-1a
	10	0,4742	-0.0333	
	100	0,3718	0.1333	
	1000	0,4558	-0.0500	
	5	0,3878	-0.1167	NO/Dectin-1b
	10	0,4742	-0.0333	
	100	0,4742	-0.0333	
	1000	0,3389	-0.1667	
Resillience	5	0,3540	0,1500	IL-6/Dectin-1a
	10	0,3389	0,1667	
	100	0,1456	0,4000	
	1000	0,0664	0,5500	
	5	0,1348	0,4167	IL-6/Dectin-1b
	10	0,0888	0,5000	
	100	0,2183	0,3000	
	1000	**0,0294**	0,6667	
	5	0,1456	0,4000	TNF-α/Dectin-1a
	10	0,2050	0,3167	
	100	0,1250	0,4333	
	1000	0,1681	0,3667	
	5	0,0738	0,5333	TNF-α/Dectin-1b
	10	0,0809	0,5167	
	100	0,1149	0,4500	
	1000	0,0540	0,5833	
	5	0,1456	0,4000	Lactate/Dectin-1a
	10	0,0969	0,4833	
	100	**0,0294**	0,6667	
	1000	0,0738	0,5333	
	5	0,1681	0,3667	Lactate/Dectin-1b
	10	0,0969	0,4833	
	100	0,0888	0,5000	
	1000	0,1348	0,4167	
	5	0.2603	-0.2500	NO/Dectin-1a
	10	0.1927	-0.3333	
	100	0.0888	0.5000	
	1000	**0,0030**	-0.8500	
	5	0,1348	-0.4167	NO/Dectin-1b
	10	0,0738	-0.5333	
	100	0,1456	-0,4000	
	1000	**0,0041**	-0.8333	
